# Lightweight Object Detection Ensemble Framework for Autonomous Vehicles in Challenging Weather Conditions

**DOI:** 10.1155/2021/5278820

**Published:** 2021-10-07

**Authors:** Rahee Walambe, Aboli Marathe, Ketan Kotecha, George Ghinea

**Affiliations:** ^1^Symbiosis Institute of Technology, Symbiosis International University, Pune, India; ^2^Symbiosis Centre for Applied Artificial Intelligence, Symbiosis International University, Pune, India; ^3^Brunel University, London, UK

## Abstract

The computer vision systems driving autonomous vehicles are judged by their ability to detect objects and obstacles in the vicinity of the vehicle in diverse environments. Enhancing this ability of a self-driving car to distinguish between the elements of its environment under adverse conditions is an important challenge in computer vision. For example, poor weather conditions like fog and rain lead to image corruption which can cause a drastic drop in object detection (OD) performance. The primary navigation of autonomous vehicles depends on the effectiveness of the image processing techniques applied to the data collected from various visual sensors. Therefore, it is essential to develop the capability to detect objects like vehicles and pedestrians under challenging conditions such as like unpleasant weather. Ensembling multiple baseline deep learning models under different voting strategies for object detection and utilizing data augmentation to boost the models' performance is proposed to solve this problem. The data augmentation technique is particularly useful and works with limited training data for OD applications. Furthermore, using the baseline models significantly speeds up the OD process as compared to the custom models due to transfer learning. Therefore, the ensembling approach can be highly effective in resource-constrained devices deployed for autonomous vehicles in uncertain weather conditions. The applied techniques demonstrated an increase in accuracy over the baseline models and were able to identify objects from the images captured in the adverse foggy and rainy weather conditions. The applied techniques demonstrated an increase in accuracy over the baseline models and reached 32.75% mean average precision (mAP) and 52.56% average precision (AP) in detecting cars in the adverse fog and rain weather conditions present in the dataset. The effectiveness of multiple voting strategies for bounding box predictions on the dataset is also demonstrated. These strategies help increase the explainability of object detection in autonomous systems and improve the performance of the ensemble techniques over the baseline models.

## 1. Introduction

The field of object detection (OD) has evolved from the conceptualization of innovative algorithms to becoming an integral part of applications in the industry. The adoption of object detection in countless real-life applications has been made possible due to the advancement of detection algorithms and the increasing computational capabilities of processors. From surveillance systems to scene understanding and face detection, object detection is being leveraged to assist humans through intelligent analytics and by automating arduous tasks. A recent application of object detection that garners interest is autonomous vehicles due to the need for fast and accurate detectors for navigation through traffic and urban environments.

In recent years, the rapid advancement of self-driving cars has transformed their image from futuristic vehicles far ahead of our time to a part of an imaginable reality. The diversity of features boasted by these vehicles is increasing day by day, with special emphasis on the interpretability of the car's decisions, ethical considerations, and overall safety [1, 2]. Designed using multiple levels of automation, the self-driving cars now can navigate through real-life traffic scenarios, avoid obstacles and pedestrians, obey driving rules, and park in vacant lots [3, 4]. To support all these functionalities, the vehicles need to perceive their surroundings as a human being would, taking into consideration the components in their environment, their relative speeds, position of the car on the road, potential hazards, road signs, traffic signals, and many more factors, enabled by their artificial sight or computer vision. These data are then collected and processed to make decisions and guide the car with all the operations needing to be accurate, computationally efficient, and explainable [5, 6]. As autonomous driving systems are improving daily, the expectation from their decision-making algorithms is vast. Not only do their decisions need to be accurate and robust, however, but they also need to be transparent and explainable while being ethically considerate [7]. One of the approaches towards this is employing robust object detection algorithms capable of the traffic scenario, outline probable navigation courses, and select one of them for execution.

The primary function of an intelligent system powering an autonomous car is the quick and accurate identification of objects in the car's immediate environment in diverse scenarios, locations, weather conditions, lighting conditions, and time. These objects may include commonplace elements like cars, pedestrians, buses, and trucks to miscellaneous elements like fallen trees, oil spills, boulders, and injured animals. The application of computer vision for this purpose has been delivered through highly accurate deep learning models trained on diverse datasets. However, there are multiple challenges, including the system's ability to function under adverse conditions, which include difficult weather conditions like rain, fog, storms, mist, and snow. As these cars rely on input from sensors, the images captured under these conditions contain unseen image corruptions and often produce erratic object detection results due to unclear object outlines and obstructed vision. The way object autonomous vehicles perceive their input differs greatly from the human gaze, and bridging this gap to design robust systems is an important challenge in computer vision that is attempted in this work.

## 2. Related Work

Leveraging computer vision for self-driving cars has evolved with the expanding requirements and research in the field and is now spread across several tasks, including vehicle detection, anomaly detection, trajectory prediction, object classification, path planning, collision avoidance, and modeling traffic rules [1, 2]. As most of these systems are usually tested under simulations, the development and training under complex scenarios can be simulated using a variety of techniques, including modeling traffic using inspiration from the theory of multiagent systems, blocking and overtaking scenarios using RC cars, and an autoencoder trained with generative adversarial costs coupled with a recurrent neural network transition model [8–11].

For avoiding obstacles and navigating through complicated traffic scenarios, efficient object detection is an important challenge for autonomous vehicles, and in this study, object detection in challenging weather conditions using the ensemble algorithm is proposed. In this section, object detection for autonomous driving is surveyed, and then the overall progress of object detection using ensemble techniques.

### 2.1. Object Detection

As autonomous vehicles respond to real-time events by understanding the scenes provided to the system through input devices like sensors, the feed from these devices can be processed for different tasks under diverse scenarios. An autonomous vehicle must be able to detect distinct objects in its surroundings like pedestrians, cars, and signs. The use of deep learning to perform object detection has been successful on several benchmark datasets and competitions like ImageNet Large Scale Visual Recognition Challenge and LiDAR data [12–14]. The active development of reliable and diverse pedestrian datasets for these models is of equal importance, and over time several datasets have been introduced, including INRIA [15], ETH [16], TUD-Brussels [17], and KITTI [18].

The datasets have been used for several autonomous vehicle-specific tasks, including 2D object detection, 3D object detection, pedestrian tracking, anomaly detection, and collision avoidance. In 2D object detection, the use of single-stage and double-stage detectors is very popular due to their high accuracy and speed, including models like YOLO, SSD, RetinaNet, R-CNN, and R-FCN. [19–28]. The double-stage detectors were able to perform better in object detection; however, the single-stage detector performed faster. In 3D object detection, additional information about the object's size and location can be leveraged to create smarter navigation systems, and the progress in 3D detectors is gaining momentum [29]. Robust models like VoxelNet, PointNet, and RoarNet were able to process 3D sensory data, combined video, and LiDAR information [30–32]. The popular methods of 3D detection can be roughly classified as Monocular Image-Based Methods, Monocular Image-Based Methods, and Fusion-Based Methods, which work by extrapolating 2D bounding boxes, generating the 3D representation of the point cloud fusing front view images and point clouds, respectively [29, 33, 34]. However, these methods are computationally expensive and require more time to execute as compared to 2D detectors. This study focuses on the performance improvement of 2D detection models and 2D data augmentation techniques.

### 2.2. Ensemble Deep Learning

The accuracy of detectors can be increased by combining CNN models, which is called an “ensemble” and can be applied to the field of autonomous driving as well. Tested on datasets like COCO and Pascal VOC [35, 36], combining SOTA models has outperformed the individual detectors in object datasets [37–39]. Ensemble models can be applied in cases with large volumes of data using combination rules or insufficient data using bootstrapping [40]. One of the main reasons that ensemble algorithms are gaining popularity is their ability to reduce both the variance and bias of learning algorithms by solving the statistical, representational, and computational problem [41].

In particular, for autonomous driving, remarkable results, particularly cone, pedestrian, and box detection for Advanced Driver Assistance Systems, were achieved through ensemble methods [42]. Thus, object detection for self-driving cars presents an important challenge, which has been tackled using various ensemble techniques, including a multispectral ensemble detection pipeline, a scalable production system for active learning, and a soft-weighted-average method for vehicle detection [43–45]. The work in this paper has been based on the algorithms proposed in [46, 47] for ensembling detectors and employing voting strategies for object detection, which was able to deliver a 10% improvement from the base models.

### 2.3. Object Detection for Resource-Constrained Devices

Creating efficient systems that are computationally efficient, consume lower power, and counter the limitations of hardware without compromising the quality of the computer vision results is one of the greatest challenges faced while designing self-driving cars. Researchers have proposed optimizing object detection through different methods, with the compression of deep learning models, reducing the computational complexity of models, and knowledge distillation among these numerous methods. Creating lightweight CNN architectures has been the focus of many works, including some state-of-the-art detectors like AlexNet [48] and hybrid approaches that achieve efficiency by combining feature extractors with neural networks [49]. Quantization [50], network pruning [51], compression [52], and efficient network design [53] were proposed for computer vision tasks on resource-constrained devices [54]. Several detectors were also proposed as efficient modifications of original networks, including Faster R-CNN, which reduced the object detection time of the original R-CNN model to less than half a second [49, 55]. Some works also proposed using Markov Decision Process frameworks for object detection [56, 57] and tracking [58]. Utilizing transfer learning for enhancing the efficiency of systems soon showed potential, as it used the knowledge from pretraining to save computational time in training models on large datasets. Several transfer learning approaches [59] gathered attention for their efficiency, including parameter transfer [60–64] and feature-representation transfer [65–69]. Neural architecture search (NAS) [70] introduced a faster method of finding efficient models using RL and showed progress on object detection [71–74]. Several works also propose boosting the performance of neural network classifiers for this task including methods like retrainable and online retrainable neural networks for nonstationary image and video data [75, 76] which work by retraining networks for enhancing application specific performance.

This work introduces the application of a two-layer lightweight ensemble framework, proposed in [47] originally for object detection in drone imagery using transfer learning, which achieved highly efficient performance in object detection tasks using pretrained models. In previous studies, the framework has also shown superior performance in detecting pedestrians in the wild [77]. A key challenge to object detection by both drones and self-driving vehicles is the limitation of hardware resources and the computational complexity of deep learning models. Therefore, designing lightweight and efficient architectures for guidance systems is one of the key focus points of researchers in the age of self-driving cars. Targeting low power consumption, minimal memory utilization, speed, efficiency, countering the limitations of hardware, and utilizing available resources efficiently while not compromising on the quality of the computer vision models requires innovation in model architectures. This paper attempts to tackle this challenge by leveraging a two-layer ensemble framework that utilizes pretrained models, transfer learning, and voting strategies to aid object detection in resource-constrained devices, which is particularly useful for self-driving cars.

In summary, the primary contributions of this paper are as follows:

1. Identification of the most effective data augmentation techniques for limited autonomous vehicles datasets typically used for object detection under adverse weather conditions of rain, mist, storms, and fog through experimentation.
2. Application of an ensemble framework combining single-stage and two-stage deep learning models for object detection. We compare the performance of ensembled models with the baseline models for object detection in corrupted images affected by adverse weather conditions of rain, mist, storms, haze, and fog.
3. Use of transfer learning to re-use the pretrained baseline models for faster processing which can prove suitable for limited resource devices in real-world applications.
4. Application of consensus, affirmative, and unanimous voting strategies for ensemble combination and studying their effects on the overall prediction accuracy.

## 3. Methodology

### 3.1. Ensemble Framework

**Figure 1:**
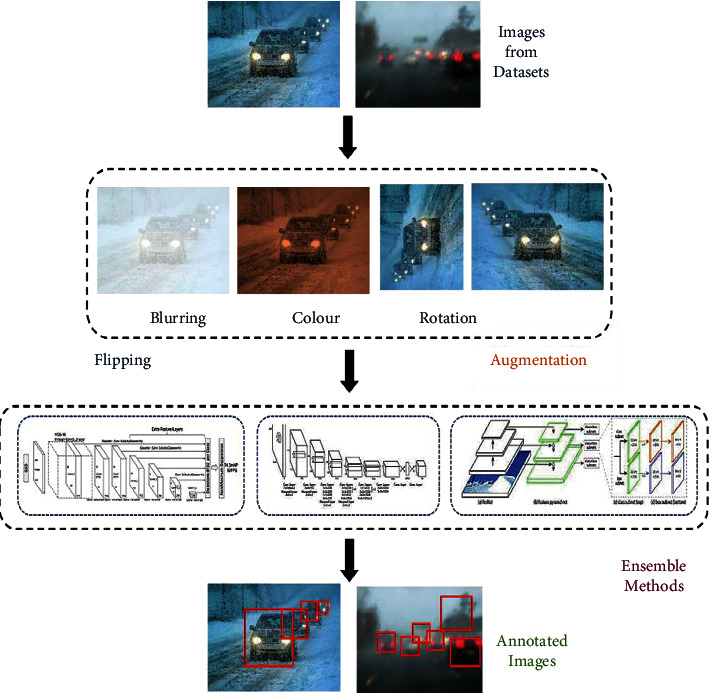
Ensemble pipeline from raw image to annotated image.

Figure 1 shows the entire pipleline of our proposed approach. It primarily consists of two components, namely, dataset collection and preprocessing including the dataset augmentation and ensembling followed by the annotated outcome.

#### 3.1.1. Ensemble Strategy

The ensemble framework applies the ensemble algorithm at 2 layers, single-model augmentation level and multimodel level. First, the algorithm combines predictions over different augmentation techniques at the single-model level and combines the predictions according to voting strategy. Then, at the multimodel level, the algorithm combines the best predictions of all the models using the voting strategy.

The ensemble algorithm works by taking a list of detections for an input image where each detection comes from the individual detectors as their outputs. The list is then flattened, and the elements are grouped based on the overlapping of bounding boxes and the corresponding classes, determined using the IoU metric [78]. For example, for two bounding boxes, *b*1 and *b*2, the overlapped region is calculated by the following formula:

(1) $$\begin{array}{c} \mathrm{IoU}\left(b1,b2\right)=\mathrm{area}\left(b1\cap b2\right)\mathrm{area}\left(b1\cup b2\right). \end{array}$$

This measure is employed to group the elements, producing, as a result, a list with the IoU threshold of 0.5. Each element of this list is focused on a particular region of the image. The size of the element determines whether the algorithm considers whether such a region contains an object and the voting strategies discussed ahead in this section. The predictions of pretrained models are combined using voting strategies, and the data augmentation boosts the results. The significant boost in efficiency is due to the complete elimination of time spent in training the model on data or combining models as the pretrained models are ensembled irrespective of the underlying algorithm and have been trained on standard openly available datasets.

#### 3.1.2. Data Augmentation

Data augmentation is an important performance-boosting technique in object detection. In this study, data augmentation was needed to improve the detectors' performance under multiple weather conditions. In addition, by increasing the diversity of the input image set, the predictions collected over different models and voting strategies were more accurate and could detect objects in dissimilar images. To improve the model performance, four different categories of data augmentation are chosen. Finally, the best one is selected for the final experiments using the score on the baseline model.

1. Color augmentation: three color augmentation techniques, raising the green channel, raising the blue channel, raising the hue of the image, were selected, and the observed performance of the models on the augmented data was recorded.
2. Rotation augmentation: four rotation augmentation techniques, rotate by 10 degrees, rotate by 90 degrees, rotate by 180 degrees, and rotate by 270 degrees for the image, were selected, and the observed performance of the models on the augmented data was recorded.
3. Flipping augmentation: four flipping augmentation techniques, vertical flip, horizontal flip, were selected along with one blurring augmentation, and the observed performance of the models on the augmented data was recorded.
4. Blurring augmentation: four blurring augmentation techniques, average blurring, bilateral blurring, Gaussian blurring, and basic blurring, were selected, and the observed performance of the models on the augmented data was recorded.

#### 3.1.3. Models

As discussed above in the related work, many 2D detectors have been introduced for object detection in traffic scenarios. Both single-stage and two-stage detectors have shown good results in the past for object detection, with their respective advantages and disadvantages. For this study, three baseline deep learning models were selected for ensembling. These models have been tested for object detection on other datasets effectively in the past and have shown good results for the OD task. Additionally, they provide a combination of single-stage and two-stage methods.

- RetinaResnet50 [79]: RetinaNet is one of the best single-stage object detectors that work well with small and densely packed objects in diverse scenarios. It uses Feature Pyramid Networks, which combines low-resolution semantically strong features with high-resolution semantically weak features and focal loss, which works on correcting wrongly classified examples making it robust for pedestrian detection. This model was trained on the COCO dataset [35].
- Yolov3 [21]: this model is an improved version of the original Darknet model, with 53 layers stacked onto the architecture. It shows improved performance than the previous Darknet-19 and is three times faster than the SSD. This model was trained on the VOC dataset [36].
- SSDResnet [22]: this is one stage of object detection. Single Shot Multibox Detector network with the inside VGG16 replaced with a ResNet50 network. This model was trained on the VOC dataset [36].

#### 3.1.4. Voting Strategies

1. Affirmative: if a single detector of the given set of detectors predicts that a region contains an object, the strategy deems this detection as valid.
2. Consensus: only if most of the initial detectors of the given set of detectors agree to consider that a region contains an object, the detection is valid.
3. Unanimous: all the detectors of the given set of detectors must agree to consider that a region contains an object.

**Figure 2:**
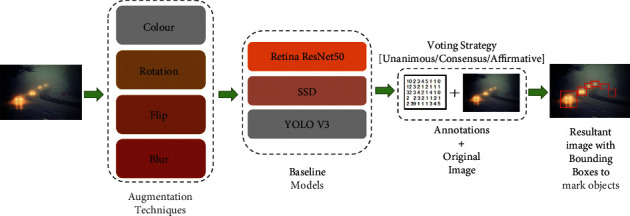
Ensemble framework for object detection.

Figure 2 shows the ensemble framework consisting of the various techniques considered for data augmentation, the baseline detectors along with the ensembling with various voting strategies, and the final outcome of the framework.

### 3.2. Data Description

There is a great need for datasets under adverse conditions like poor weather, poor resolution, and multiple scaled objects for creating robust autonomous driving systems. To meet this demand, a large vehicle detection dataset in adverse weather nature named DAWN was created in 2020 to assist in object detection, segmentation, and image processing applications [80, 81]. The DAWN dataset is a collection of 1000 images from real-traffic environments, collected from 4 adverse weather conditions: fog, snow, rain, and sandstorms. This study focuses on the fog and rain conditions that contain images under fog, rain, mist, haze, and stormy weather.

**Figure 3:**
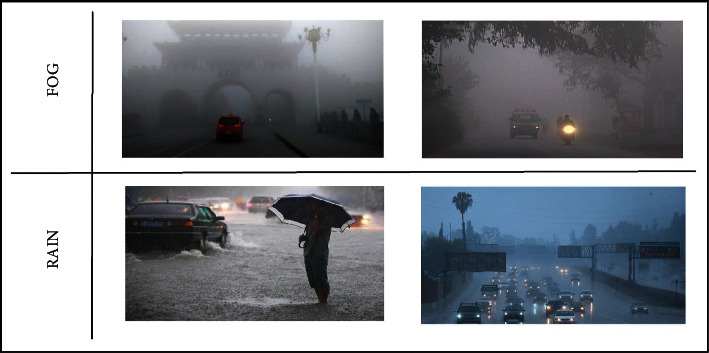
DAWN dataset sample with rain and fog weather conditions.

For this study, 500 images of driving conditions under rain, mist, haze, and fog are used to test the ensemble models, which form a total of 1500 samples after the data augmentation. The dataset contains significant variation in vehicle category, size, orientation, pose, illumination, position, and occlusion. The annotations for the DAWN dataset contain two categories of objects, vehicles and humans, which cover the vehicles' classes (e.g., car, bus, truck, motorcycle, and bicycle) and human classes such as cyclist and pedestrian. The size of the input images is 1,280 × 856‬ pixels.‬‬‬‬‬‬‬‬‬‬‬‬‬‬‬‬‬‬ Figure 3 shows sample images from the DAWN dataset.‬‬‬‬‬‬‬‬‬‬‬‬‬‬

## 4. Results and Discussion

### 4.1. Experiments

**Figure 4:**
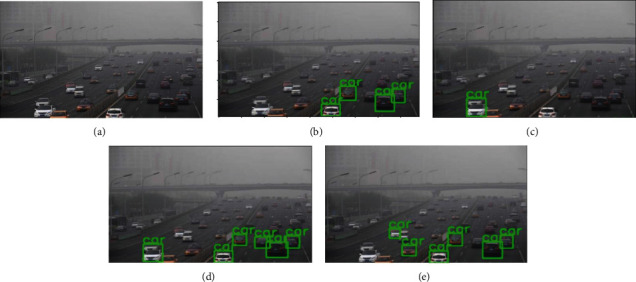
Performance of multiple augmentation techniques on DAWN dataset. (a) Original image. (b) Blur augmentation. (c) Rotation augmentation. (d) Flip augmentation. (e) Color augmentation.

**Figure 5:**
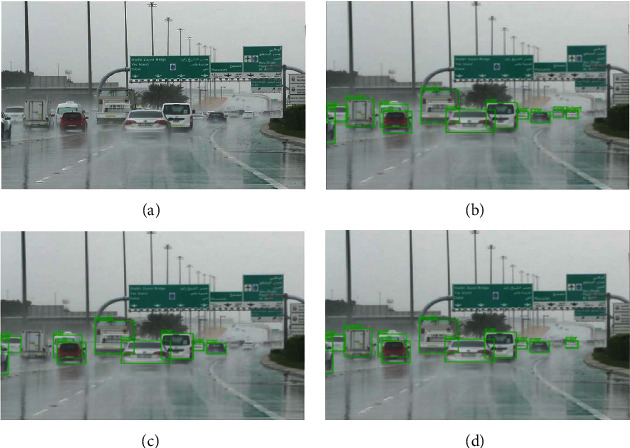
Performance of voting strategies: affirmative, consensus, and unanimous, on DAWN dataset. The affirmative strategy appears to capture both medium- and small-scale objects in the image. (a) Original image. (b) Affirmative strategy. (c) Unanimous strategy. (d) Consensus strategy.

**Figure 6:**
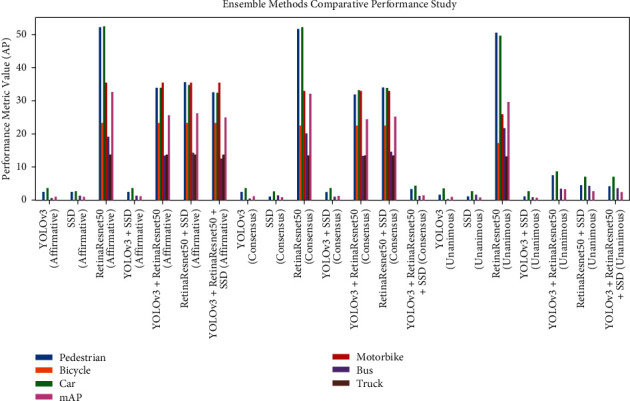
Performance of all models measured using class-wise AP. The RetinaResnet50 model shows consistently good results over multiple voting strategies.

The experiments for this study were carried out in stages, examining and combining the results from each stage to move on to the next and eventually obtain the best results. For each stage, the results were measured in terms of the AP (average precision) for each of the six classes and the overall AP for all classes. The first stage was testing the baseline object detection model on the original dataset without any augmentation. The corresponding results with and without augmentation are shown in Table 1. The next stage was using one baseline model (SSD) and one voting strategy (affirmative) and running the detection on the four different types of augmentation to find the best data augmentation technique. Finally, after finding the best augmentation technique, it is applied to the three baseline models and four ensemble models with all three strategies: consensus, affirmative, and unanimous, to find the best performing model and strategy overall. The corresponding results are shown in Table 2. Figures 4–6 show the performance of the ensemble models and augmentation techniques.

The performance of the SSD model on different classes after augmentation provides interesting insight on the model and class difficulty. In spite of the model's training data being VOC which contains bicycles and motorbikes as objects, the model is unable to detect bicycles and motorbikes under difficult weather conditions. This may be due to the blurring effect when these smaller vehicles in high speed are photographed in bad weather. This drawback does not appear for pedestrians due to their slow pace. The training data do not contain trucks as a separate class, and thus the pretrained model cannot identify any trucks in the target data. This is one drawback of the ensemble framework and to be eliminated the classes of target data should be covered in training data for robust performance across all classes.

The flip and color augmentation can be observed to produce the maximum correct predictions in adverse weather conditions, as seen in Figure 4.

The affirmative voting strategy can capture predictions from the models even for the smallest vehicles under adverse weather conditions, as seen in the top right corner of the affirmative strategy output image in Figure 5.

### 4.2. Comparison with Previous Works

Table 3 compares the developed approach with the existing work on the DAWN dataset [82, 84]. The previous works present an array of detectors [84] that obtain the state-of-the-art performance detecting objects in the dataset under adverse weather conditions. One of the top detectors was the Faster R-CNN with several RPNs that could attain 89.48% mAP when trained and tested on subsets of the DAWN dataset. However, as the proposed framework uses pretrained models trained without using the DAWN dataset, the results are compared with other models that perform detections without training on the data, which have been presented in [82]. These models were trained on the ImageNet dataset [12] and fine-tuned on the “clear” split of BDD100k-cls [83]. The four models presented here are ResNet50 backbones used in Faster R-CNN and show the optimal performance of 25.8% mAP using ensemble (AMDA, AMDA), which is a two-member ensemble of the state-of-the-art AMDA model trained with AugMix and DeepAugment. The top-performing ensemble model RetinaResnet50 with affirmative voting strategy is able to attain 32.75% mAP which outperforms the previous models by 6.95% mAP when exposed to testing data with unseen corruptions of rainy and foggy weather conditions.

### 4.3. Discussion

After conducting the experiments, baseline models, augmentation techniques, ensemble models, and voting strategies were observed. The performance of baseline RetinaResnet50 outperforms all the other detectors by over 30% AP and is consistently contributing good results. The training samples are affecting the performance as well, as the models trained on VOC dataset are unable to compete with RetinaResnet50 trained on COCO dataset for this task. Color-based augmentation performed the best, with a 0.2% mAP increase over the second-best blurring augmentation technique for data augmentation. The difference in performance before and after augmentation was not very significant, with a maximum of 0.72% boost in AP. Selecting the color augmentation technique, testing the performance of 7 models was carried out next. RetinaResnet50 with affirmative voting strategy consistently performed better than regular and ensemble models, and the consensus and unanimous strategies for the same model with the highest 32.75% AP. However, for the bus OD, RetinaResnet50 with unanimous strategy gave better results than the affirmative strategy by 2%. For bicycles, motorbikes, and trucks, Yolov3 + RetinaResnet50, RetinaResnet50 + SSD, and RetinaResnet50 + SSD + Yolov3 with the affirmative strategy were able to match the performance of the RetinaResnet50. Overall, out of the three strategies, affirmative performed the best. For object detection in challenging scenarios, the best performing ensemble model RetinaResnet50 can outperform the other models which were trained on datasets other than the DAWN dataset in terms of average precision for rain and fog conditions. Its performance cannot match the SOTA trained on the DAWN dataset; however, it demonstrates a good performance for object detection. For pedestrian detection, the best performing model RetinaResnet50 can achieve 41.41% AP and utilizes pretrained models with the DAWN dataset. Through the test time augmentation and efficient voting strategies, the lightweight framework produces instant predictions on the target data and shows good performance as well. In real time, this method can be used to detect objects captured in feed according to the prediction voting strategy used. This framework presents a viable solution to combating computational complexity for resource-constrained devices while simultaneously providing robust predictions.

## 5. Conclusion

This study presented the use of an efficient ensemble algorithm for object detection on a dataset for autonomous vehicles under adverse weather conditions. When combined with voting strategies and data augmentation, the algorithms performed best in detecting pedestrians and vehicles. The affirmative strategy combined the detection results most effectively and demonstrated the best results out of all the voting strategies. The ability of RetinaResnet50 to detect objects under adverse weather conditions shows promise for the future of robust FPN-based single-stage detectors. The key feature of this study is the object detection performance of the ensembled models via transfer learning of pretrained baseline models on data with unseen image corruptions. The effectiveness of this framework for object detection in the dataset with image corruptions due to rainy and foggy weather conditions is demonstrated. The lightweight architecture can be deployed on any resource-constrained device as the predictions are directly generated without delay and can be used for continuous video streams or data.

The future work involves testing the performance of this algorithm in detecting miscellaneous objects like traffic signs, rocks, and obstacles and testing their ability to differentiate between similar objects like bicycles, bikes, and tricycles. These experiments can be extended to other autonomous driving datasets in challenging scenarios. The current framework carries out detection in the absence of temporal information which can be incorporated in the future for tracking and trajectory prediction. Testing the performance of the ensemble algorithm against adversarial attacks will also be a useful application for self-driving cars. The experiments with ensemble algorithms and data augmentation for robust object detection in challenging scenarios like fog, rainfall, and mist were successful. We hope that this will contribute to the development of reliable and safe autonomous driving systems in the future.

## Figures and Tables

**Table 1:** SSD OD results on DAWN dataset before and after augmentation.

Model	Augmentation	Class 1 (pedestrian) AP (%)	Class 2 (bicycle) AP (%)	Class 3 (car) AP (%)	Class 4 (motorbike) AP (%)	Class 5 (bus) AP (%)	Class 6 (truck) AP (%)	mAP (%)
SSD	None	1.03	0.00	2.61	0.00	1.49	0.00	0.85
SSD	Flipping	1.23	0.00	1.80	0.00	1.25	0.00	0.71
SSD	Blurring	1.25	0.00	2.16	0.00	0.99	0.00	0.73
SSD	Rotations	0.28	0.00	0.14	0.00	0.23	0.00	0.11
SSD	Color	**1.54**	0.00	**2.72**	0.00	**1.34**	0.00	**0.93**

**Table 2:** Best performing models for OD on the DAWN dataset with color augmentation.

Model	Voting strategy	Class 1 (pedestrian) AP (%)	Class 2 (bicycle) AP (%)	Class 3 (car) AP (%)	Class 4 (motorbike) AP (%)	Class 5 (bus) AP (%)	Class 6 (truck) AP (%)	mAP (%)
RetinaResnet50	Unanimous	50.53	17.19	49.71	25.93	**21.73**	13.18	29.71
RetinaResnet50	Affirmative	**52.34**	**23.29**	**52.56**	**35.51**	19.09	**13.71**	**32.75**
Yolov3 + RetinaResnet50	Affirmative	34.03	**23.29**	33.98	**35.51**	13.56	**13.71**	25.68
RetinaResnet50 + SSD	Affirmative	35.63	**23.29**	34.79	**35.51**	14.28	**13.71**	26.20
Yolov3 + RetinaResnet50 + SSD	Affirmative	32.53	**23.29**	32.51	**35.51**	12.59	**13.71**	25.03

**Table 3:** Comparison of previous models to the proposed model for OD performance on DAWN dataset [82, 83].

Models	Training dataset	Voting strategy	mAP (of rain and fog) (in %)
*Previous models (mAP is for all 4 categories)*			
Faster R-CNN with several RPNs (with Faster R-CNN backbone) [84]	DAWN	n/a	**89.48**

*Previous models*			
Standard data augmentation [82]	Pretrained on ImageNet and fine-tuned on the” clear” split of BDD100k-cls	n/a	**23.3**
AMDA [82]	Pretrained on ImageNet and fine-tuned on the” clear” split of BDD100k-cls	n/a	**25.55**
Ensemble (AMDA, AMDA) [82]	Pretrained on ImageNet and fine-tuned on the” clear” split of BDD100k-cls	n/a	**25.8**
RoHL (AMDATV-ftGauss, AMDA-ftCont) [82]	Pretrained on ImageNet and fine-tuned on the HF and LF biases.	n/a	**24.9**

*Proposed models*			
RetinaResnet50	COCO	Affirmative	**32.75**

## Data Availability

The DAWN dataset [80, 81] can be accessed at https://data.mendeley.com/datasets/766ygrbt8y/3.

## References

[1] Nyholm S., Smids J. (2016). The ethics of accident-algorithms for self-driving cars: an applied trolley problem?. *Ethical Theory and Moral Practice*.

[2] Belay N. (2015). Robot ethics and self-driving cars: how ethical determinations in software will require a new legal framework. *Journal of the Legal Profession*.

[3] Daily M., Medasani S., Behringer R., Trivedi M. (2017). Self-driving cars. *Computer*.

[4] Badue C., Guidolini R., Vivacqua Carneiro R. (2020). Self-driving cars: a survey. *Expert Systems with Applications*.

[5] Nielsen T., Sick A., Haustein S. (2018). On sceptics and enthusiasts: what are the expectations towards self-driving cars?. *Transport Policy*.

[6] Urmson C., Whittaker W. (2008). Self-driving cars and the urban challenge. *IEEE Intelligent Systems*.

[7] Holstein T., Dodig-Crnkovic G., Pelliccione P. (2018). Ethical and social aspects of self-driving cars. https://arxiv.org/abs/1802.04103.

[8] Santana E., George H. (2016). Learning a driving simulator. https://arxiv.org/abs/1608.01230.

[9] Gora P., Rüb I. (2016). Traffic models for self-driving connected cars. *Transportation Research Procedia*.

[10] Wang M., Wang Z., Talbot J., Christian Gerdes J., Schwager M. (2021). Game theoretic planning for self-driving cars in competitive scenarios. *IEEE Transactions on Robotics*.

[11] Chen S., Chen Y., Zhang S., Zheng N. (2019). A novel integrated simulation and testing platform for self-driving cars with hardware in the loop. *IEEE Transactions on Intelligent Vehicles*.

[12] Russakovsky O., Deng J., Su H. (2015). Imagenet large scale visual recognition challenge. *International Journal of Computer Vision*.

[13] Qi C. R., Liu W., Wu C., Su H., Guibas L. J. Frustum pointnets for 3d object detection from rgb-d data.

[14] Chen X., Ma H., Wan J., Li B., Xia T. Multi-view 3d object detection network for autonomous driving.

[15] Dalal N., Triggs B. Histograms of oriented gradients for human detection.

[16] Ess A., Leibe B., Van Gool L. Depth and appearance for mobile scene analysis.

[17] Wojek C., Walk S., Bernt Schiele Multi-cue onboard pedestrian detection.

[18] Geiger A., Lenz P., Urtasun R. Are we ready for autonomous driving? the kitti vision benchmark suite.

[19] Redmon J., Divvala S., Girshick R., Ali F. You only look once: unified, real-time object detection.

[20] Redmon J., Ali F. YOLO9000: better, faster, stronger.

[21] Redmon J., Ali F. (2018). Yolov3: an incremental improvement. https://arxiv.org/abs/1804.02767.

[22] Liu W., Anguelov D., Erhan D. Ssd: single shot multibox detector.

[23] Law H., Jia D. Cornernet: detecting objects as paired keypoints.

[24] Zhang S., Wen L., Bian X., Lei Z., Li S. Z. Single-shot refinement neural network for object detection.

[25] Girshick R., Donahue J., Darrell T., Malik J. Rich feature hierarchies for accurate object detection and semantic segmentation.

[26] Ren S., He K., Girshick R., Sun J. (2015). Faster r-cnn: towards real-time object detection with region proposal networks. https://arxiv.org/abs/1506.01497.

[27] Dai J., Li Y., He K., Sun J. (2016). R-FCN: object detection via region-based fully convolutional networks. https://arxiv.org/abs/1605.06409.

[28] Masmoudi M., Ghazzai H., Frikha M., Massoud Y. Object detection learning techniques for autonomous vehicle applications.

[29] Arnold E., Al-Jarrah O. Y., Dianati M., Fallah S., Oxtoby D., Mouzakitis A. (2019). A survey on 3d object detection methods for autonomous driving applications. *IEEE Transactions on Intelligent Transportation Systems*.

[30] Qi C. R., Su H., Mo K., Guibas L. J. Pointnet: deep learning on point sets for 3d classification and segmentation.

[31] Zhou Y., Tuzel O. Voxelnet: end-to-end learning for point cloud based 3d object detection.

[32] Shin K., Kwon Y. P., Tomizuka M. Roarnet: a robust 3d object detection based on region approximation refinement.

[33] Srivastava S., Jurie F., Sharma G. Learning 2d to 3d lifting for object detection in 3d for autonomous vehicles.

[34] Meng Q., Song H., Li G., Zhang Y., Zhang X. (2019). A block object detection method based on feature fusion networks for autonomous vehicles. *Complexity*.

[35] Lin T.-Y., Maire M., Belongie S. Microsoft coco: common objects in context.

[36] Everingham M., Van Gool L., Williams C. K. I., Winn J., Zisserman A. (2010). The pascal visual object classes (voc) challenge. *International Journal of Computer Vision*.

[37] Guo J., Gould S. (2015). Deep CNN ensemble with data augmentation for object detection. https://arxiv.org/abs/1506.07224.

[38] Lee J., Lee S.-K., Yang S.-I. An ensemble Method of Cnn Models for object detection.

[39] Xu J., Wang W., Wang H., Guo J. (2020). multi-model ensemble with rich spatial information for object detection. *Pattern Recognition*.

[40] Polikar R. (2012). Ensemble learning. *Ensemble Machine Learning*.

[41] Dietterich T. G. (2002). Ensemble learning. *The Handbook of Brain Theory and Neural Networks*.

[42] Wei P., Ball J., Anderson D. (2018). Fusion of an ensemble of augmented image detectors for robust object detection. *Sensors*.

[43] Wang H., Cai Y., Yu Y., Chen X., Chen L., Li Y. (2020). Soft-weighted-average ensemble vehicle detection method based on single-stage and two-stage deep learning models. *IEEE Transactions on Intelligent Vehicles*.

[44] Takumi K., Watanabe K., Ha Q., Tejero-de-Pablos A., Ushiku Y., Harada T. Multispectral object detection for autonomous vehicles.

[45] Haussmann E., Fenzi M., Chitta K. Scalable Active Learning for object detection.

[46] Casado-García Á., Heras J. Ensemble methods for object detection.

[47] Walambe R., Marathe A., Kotecha K. (2021). Multiscale object detection from drone imagery using ensemble transfer learning. *Drones*.

[48] Krizhevsky A., Sutskever I., Hinton G. E. ImageNet classification with deep convolutional neural networks.

[49] Girshick R. Fast r-cnn.

[50] Guo Y. (2018). A survey on methods and theories of quantized neural networks. https://arxiv.org/abs/1808.04752.

[51] Lin S., Ji R., Li Y., Deng C., Li X. (2019). Towards compact convnets via structure-sparsity regularized filter pruning. https://arxiv.org/abs/1901.07827.

[52] Han S., Pool J., Tran J., Dally W. J. (2015). Learning both weights and connections for efficient neural networks. https://arxiv.org/abs/1506.02626.

[53] Sandler M., Howard A. G., Zhu M., Zhmoginov A., Chen L. (2018). Inverted residuals and linear bottlenecks: mobile networks for classification, detection and segmentation. https://arxiv.org/abs/1801.04381.

[54] Plastiras G., Siddiqui S., Kyrkou C., Theocharides T. Efficient embedded deep neural-network-based object detection via joint quantization and tiling.

[55] Ren S., He K., Girshick R., Sun J. (2015). Faster r-cnn: towards real-time object detection with region proposal networks. *Advances in Neural Information Processing Systems*.

[56] Paletta L., Fritz G., Seifert C. Q-learning of sequential attention for visual object recognition from informative local descriptors.

[57] Karayev S., Fritz M., Darrell T. Anytime recognition of objects and scenes.

[58] Xiang Y., Alahi A., Savarese S. Learning to track: online multiobject tracking by decision making.

[59] Pan S. J., Yang Q. (2009). A survey on transfer learning. *IEEE Transactions on Knowledge and Data Engineering*.

[60] Lawrence N. D., Platt J. C. Learning to learn with the informative vector machine.

[61] Bonilla E., Chai K. M., Williams C. Multi-task Gaussian process prediction.

[62] Schwaighofer A., Tresp V., Yu K. Learning Gaussian process kernels via hierarchical bayes.

[63] Evgeniou T., Pontil M. Regularized multi-task learning.

[64] Gao J., Fan W., Jiang J., Han J. Knowledge transfer via multiple model local structure mapping.

[65] Dai W., Xue G., Yang Q., Yu Y. “Co-Clustering based classification for out-of-domain documents.

[66] Ando R. K., Zhang T. A high-performance semi-supervised learning method for text chunking.

[67] Blitzer J., McDonald R., Pereira F. Domain adaptation with structural correspondence learning.

[68] Daume H. Frustratingly easy domain adaptation.

[69] Argyriou A., Evgeniou T., Pontil M. Multi-task feature learning.

[70] Zoph B., Le Q. V. (2016). Neural architecture search with reinforcement learning. https://arxiv.org/abs/1611.01578.

[71] Ghiasi G., Lin T.-Y., Le Q. V. Nas-fpn: learning scalable feature pyramid architecture for object detection.

[72] Chen Y., Tong Y., Zhang X., Meng G., Pan C., Sun J. (2019). Detnas: neural architecture search on object detection. https://arxiv.org/abs/1903.10979.

[73] Wang N., Gao Y., Chen H. (2019). Nas-fcos: fast neural architecture search for object detection. https://arxiv.org/pdf/1906.04423.

[74] Chen B., Ghiasi G., Liu H. Mnasfpn: learning latency-aware pyramid architecture for object detection on mobile devices.

[75] Doulamis A. D., Doulamis N. D., Kollias S. D. (2000). On-line retrainable neural networks: improving the performance of neural networks in image analysis problems. *IEEE Transactions on Neural Networks*.

[76] Doulamis A. D., Doulamis N. D., Kollias S. D. Retrainable neural networks for image analysis and classification.

[77] Marathe A., Walambe R., Kotecha K. Evaluating the performance of ensemble methods and voting strategies for dense 2D pedestrian detection in the wild.

[78] Rosebrock A. (2016).

[79] Lin T.-Y., Goyal P., Girshick R., He K., Dollar P. Focal loss for dense object detection.

[80] Kenk M. A., Hassaballah M. (2020). DAWN: vehicle detection in adverse weather nature dataset. https://arxiv.org/abs/2008.05402.

[81] Kenk M. (2020). Dawn. *Mendeley Data*.

[82] Saikia T., Schmid C., Brox T. (2021). Improving robustness against common corruptions with frequency biased models. https://arxiv.org/abs/2103.16241.

[83] Yu F., Chen H., Wang S. Bdd100k: a diverse driving dataset for heterogeneous multitask learning.

[84] Ghosh R. (2021). On-road vehicle detection in varying weather conditions using faster R-CNN with several region proposal networks. *Multimedia Tools and Applications*.

